# Probiotic supplementation in preterm infants does not affect the risk of retinopathy of prematurity: a meta-analysis of randomized controlled trials

**DOI:** 10.1038/s41598-017-13465-2

**Published:** 2017-10-12

**Authors:** Giacomo Cavallaro, Eduardo Villamor-Martínez, Luca Filippi, Fabio Mosca, Eduardo Villamor

**Affiliations:** 1 Neonatal Intensive Care Unit, Department of Clinical Sciences and Community Health, Fondazione IRCCS Cà Granda Ospedale Maggiore Policlinico, Università degli Studi di Milano, Milan, 20122 Italy; 2 Department of Pediatrics, Maastricht University Medical Center (MUMC+), School for Oncology and Developmental Biology (GROW), Maastricht, 6202 AZ Netherlands; 3 Neonatal Intensive Care Unit, Medical and Surgical Feto-Neonatal Department, “A. Meyer” University Children’s Hospital, 50139 Florence, Italy

## Abstract

Retinopathy of prematurity (ROP) is a vascular disorder of the developing retina in preterm infants and is a leading cause of childhood blindness. Perinatal infection plays a pathogenic role in ROP. Probiotic supplementation reduces the risk of late onset sepsis (LOS) in preterm infants but it remains to be determined whether this reduction translates into a reduction of other complications. We conducted a systematic review and meta-analysis to evaluate the possible role of probiotics in altering the risk of ROP. Eleven randomized controlled trials (4250 infants; probiotics: 2121) were included in the meta-analysis that showed a significantly decreased rate of LOS with a risk ratio (RR) of 0.807 and a 95% confidence interval (CI) of 0.705 to 0.924 (P = 0.010; fixed effects model) but could not demonstrate a significant effect of probiotics on any stage ROP (RR 1.053, 95% CI 0.903 to 1.228, P = 0.508, 4 studies), or severe ROP (RR 0.841, 95% CI 0.666 to 1.063, P = 0.148, 9 studies). Meta-regression did not show any significant association between the RR for LOS and the RR for severe ROP. In conclusion, our results suggest that infection prevention by probiotics does not affect the risk of developing ROP in preterm infants.

## Introduction

Retinopathy of prematurity (ROP) is a vascular disorder of the developing retina in preterm infants and is a leading cause of childhood blindness^1–7^. ROP progresses in two phases. The first phase begins with delayed retinal vascular growth after birth and partial regression of existing vessels, followed by a second phase of hypoxia-induced pathological vessel growth^8^. Low gestational age (GA), low birth weight (BW), and supplemental oxygen therapy following delivery have consistently been associated with ROP^1–7^. However, ROP is a multifactorial disease and multiple other modifiable clinical factors have been associated with an increased risk of ROP. These include, among others, hypoxia, hypercapnia, hyperglycaemia, exposure to blood transfusions, poor postnatal weight gain, and perinatal infection/inflammation^1–6,9–14^. While prevention of ROP would be best aimed at reducing preterm birth, postnatal preventive efforts are directed at reducing the other stressors that may lead to injury of the developing retinal vessels^15^.

In a very recent systematic review, Fang *et al*. analysed the effectiveness of oxygen saturation targeting, nutritional interventions, blood transfusion management, and infection prevention on the incidence of ROP^1^. They found that lower oxygen saturation targets reduced the risk of developing any stage ROP and severe ROP but increased mortality. In addition, aggressive parenteral nutrition reduced the risk of any stage ROP but not severe ROP. Supplementation of vitamin A, E, or inositol and breast milk feeding were beneficial but the effect was not observed in randomized controlled trials (RCTs)^1^. Finally, when the authors analysed the literature on infection prevention and its effect on the incidence of ROP, they only included studies on fluconazole prophylaxis of invasive fungal infections because these studies were the only reporting sufficient numbers for quantitative analysis. Despite a reduced risk of invasive fungal infection, fluconazole prophylaxis had no significant effect on the risk of developing severe ROP^1^.

Experimental and clinical studies support the concept that modulation of intestinal microbiota in preterm infants may alter the risk of infection, and systemic inflammatory response syndrome either directly or through immune modulation^16–18^. Probiotic bacteria are live microbial supplements that colonize the gastrointestinal tract and potentially provide benefit to the host^19–21^. Recent meta-analyses showed that probiotic supplementation reduces the time to achieve full enteral feeding and the risk of developing necrotizing enterocolitis (NEC) as well as late onset sepsis (LOS) in preterm infants^22–34^. Therefore, probiotic supplementation can be considered as a method of infection prevention in this population. In addition, some probiotic strains may have antioxidant properties^35^. Interestingly, a number of RCTs of probiotic supplementation in preterm infants included data on ROP as secondary outcome. Nevertheless, systematic analysis regarding the possible effect of probiotics on preventing ROP is lacking. The objective of this systematic review and meta-analysis was to study the effect of probiotic supplementation in the incidence of ROP in preterm infants.

## Methods

A protocol was developed prospectively that detailed the specific objectives, criteria for study selection, the approach to assessing study quality, clinical outcomes, and statistical methodology. The study is reported according to the PRISMA checklist^36^.

### Data Sources and Search Strategies

A comprehensive literature search was undertaken using PubMed, EMBASE and CENTRAL (the Cochrane Central Register of Controlled Trials, The Cochrane Library) from their inception to March 1, 2016. The search terms used in the three databases were (probiotic(s) OR lactobacillus OR saccharomyces OR bifidobacterium OR streptococcus) AND (retinopathy of prematurity OR sepsis OR late onset sepsis). Language was not restricted. Additional strategies to identify studies included manual review of reference lists of key articles that fulfilled our eligibility criteria, use of the “related articles” feature in PubMed, use of the “cited by” tool in Google Scholar, and manual review of reference lists of meta-analyses on probiotics in preterm infants^22–34,37,38^.

### Eligibility Criteria and Study Selection

Investigators were divided into two groups (E.V.-M./E.V. and G.C./L.F.). Both groups searched the literature independently and assessed the eligibility of trials for inclusion in the review. Disagreements were settled by discussion and, if necessary, the other author (F.M.) was consulted. All titles and abstracts of papers identified by the search strategy were screened for relevance. At this stage, only clearly non-relevant articles were excluded. Full copies of all potentially relevant papers were obtained and texts were screened to assess eligibility for inclusion. Studies were included if they were RCTs involving the use of probiotics in preterm infants (GA <37 weeks) and reported results on ROP. Studies were excluded if they did not meet all of these inclusion criteria. Studies were reviewed to ensure that study populations did not overlap by checking subject sources and study time-frame. Where two or more studies reported on the same population, the most recent study was preferentially used (provided it reported data on ROP) to avoid duplicate data.

### Data Extraction and Assessment of Risk of Bias

The two groups of investigators extracted the data independently by using a data collection form designed for this review. Data extracted included: GA and BW of participants, patient inclusion criteria, study design (age at the first day of intervention, duration of intervention, dosage, and type of probiotic), and outcomes of interest. We defined ROP as any stage ROP or severe ROP (including stage 3 or 4, surgical, and threshold ROP). The number of cases of ROP and the number of patients analysed in each treatment group of each trial were entered into the form. Data on LOS were also extracted.

Two reviewers (G.C. and E.V.) independently assessed risk of bias in each trial by using the Cochrane “Risk of Bias Assessment Tool”^39^. For each domain (allocation sequence, allocation concealment, blinding of participants and outcome assessors, incomplete outcome data, selective outcome reporting, and other potential sources of bias) the risk was assessed as low, high, or unclear. Potential discrepancies during the data extraction process and assessment of risk of bias were resolved by discussion and consensus among all reviewers.

### Statistical Analysis

Studies were combined and analysed using comprehensive meta-analysis V 3.0 software (Biostat Inc., Englewood, NJ, USA). A fixed-effects model (Mantel–Haenszel; M-H) was used. However, analysis using random effects model was also conducted to ensure that the results and conclusions were not influenced by the type of model used for the meta-analysis. Effect size was expressed as risk ratio (RR) and 95% confidence interval (CI). Statistical heterogeneity was assessed with the Cochran’s Q statistic and by the *I* ^2^ statistic, which is derived from Q and describes the proportion of total variation that is due to heterogeneity beyond chance^40^. An *I* ^2^ value of 0% indicates no observed between-study heterogeneity, and large values show increasing between-study heterogeneity. The risk of publication bias was assessed by visual inspection of the funnel plot and using Egger test. To identify any study that may have exerted a disproportionate influence on the summary effect, we deleted studies one at a time. To explore differences between studies that might be expected to influence the effect size, we performed univariate random-effects meta-regression (method of moments)^41,42^. A probability value of less than 0.05 (0.10 for heterogeneity) was considered statistically significant.

## Results

**Figure 1:**
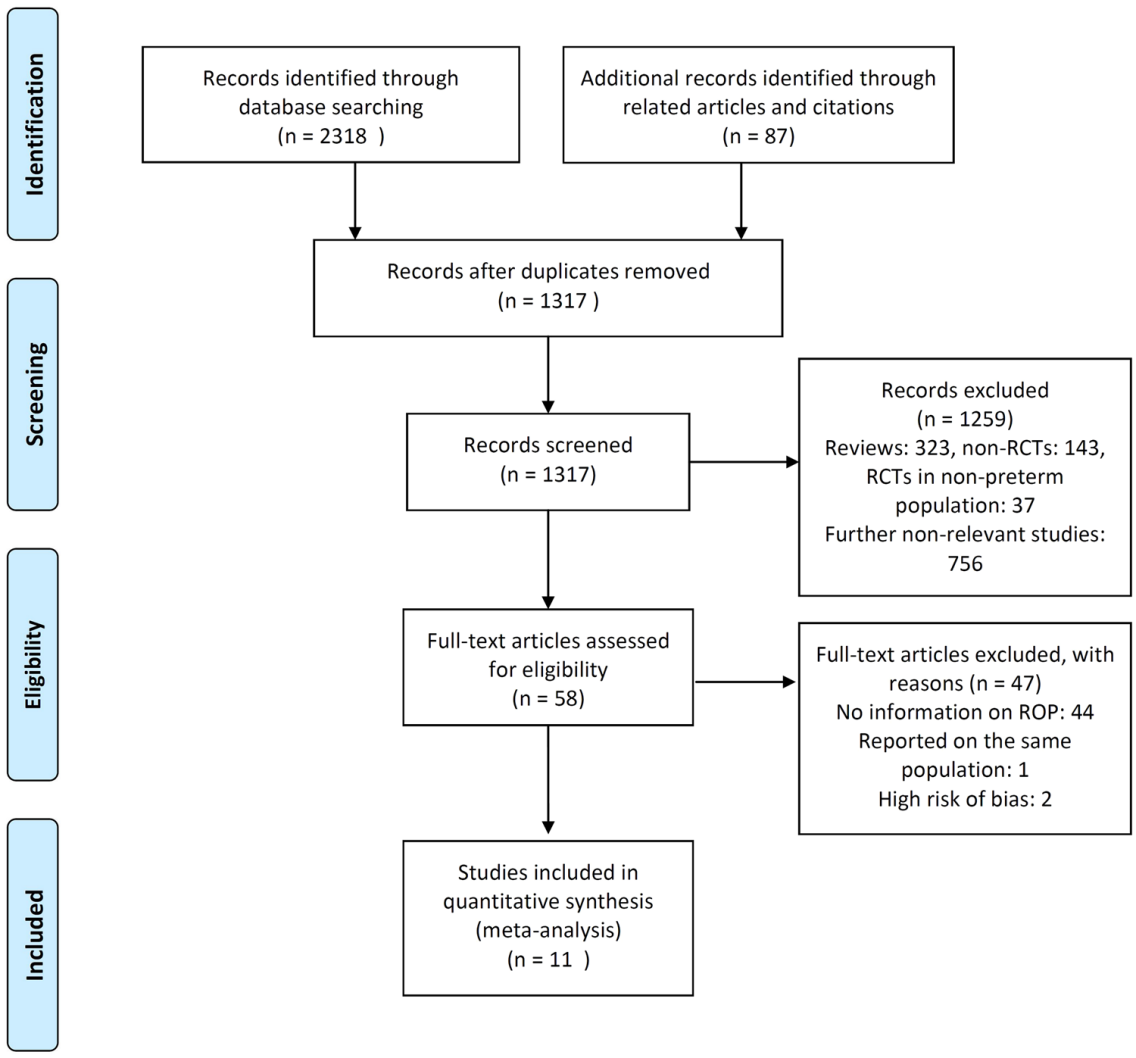
PRISMA flow diagram of search strategy and study selection.

There was no substantial disagreement between the reviewers on articles for inclusion, data extraction, and risk of bias assessment. Based on the titles and abstracts of 1317 citations, we identified 58 potentially relevant studies, 11 of which met the inclusion criteria^43–53^ (Figure 1, Table 1). Two additional studies^54,55^ met the inclusion criteria but were excluded due to the presence of unclear or high risk of bias in more than three domains (Table 2). The main characteristics of the studies are shown in Table 1. The 11 studies included 4250 infants from which 2121 infants received probiotics. Nine studies included very preterm (GA <32 weeks) and/or very low BW (<1500 g) infants^44–49,51–53^. One study included extremely low BW preterm infants (<1000 g)^43^. One study included larger preterm infants (GA <37 weeks and BW <2500 g)^50^. The included studies randomized infants to different preparations, times of initiation and duration of therapy (Table 1). Of the 11 included studies, 10^44–53^ (91%) were judged to have low risk of bias for the domain of “random sequence generation,” and 7^44–48,50,51^ (64%) were considered to have low risk of bias for “allocation concealment.” Details of the risk of bias analysis are depicted in Table 2. Additional data on percentage of cesarean section, use of antenatal corticosteroids, antenatal antibiotics, maternal infection, preterm rupture of membranes (PROM), and use of exclusive maternal milk are shown in Supplementary Table 1.

**Table 1:** Characteristics of the included studies.

Study	Inclusion criteria (BW and/or GA)	Sample size		Intervention	Duration of intervention	Primary outcome	ROP		LOS	
		Probiotics	Control				Pr	Co	Pr	Co
Al Hosni 2012^43^	BW 501–1000 g	n = 50 GA: 25.7 (1.4) BW: 778 (138)	n = 51 GA: 25.7 (1.4) BW: 779 (126)	*L*. *rhamnosus* + *B*. *infantis* vs. no probiotics	Once daily from the time of initiation of enteral feeds, until discharge or 34 wks PMA	% infants with weight <10th centile at 34 wks PMA	Any 32/44 Severe 8	Any 28/48 Severe 4	13/50	16/51
Chou 2010^44^	BW <1500 g	n = 153 GA: 28.5 (2.3) BW: 1103 (232)	n = 148 GA: 28.5 (2.3) BW: 1097 (231)	*L*. *acidophilus* + *B*. *infantis* vs. No probiotics	After d 7 of life, from the time of commencement of enteral feeds, until discharge	Death or neurodevelopmental impairment	Severe 9/153	Severe 15/148	21/153	30/148
Costeloe 2016^45^	GA <31 wk	n = 650 GA: 27.8 (2.5) BW: 1039 (312)	n = 660 GA: 27.9 (2.6) BW: 1043 (317)	*B. breve* BBG-001 vs. placebo	Commenced within 48 h of birth, until 36 wks PMA	≥ Stage 2 NEC, LOS, death	Any 160/600 Severe 23	Any 161/605 Severe 25	73/650	77/660
Demirel 2013^46^	GA ≤32 wk and BW ≤1500 g	n = 135 GA: 29.4 (2.3) BW: 1164 (261)	n = 136 GA: 29.2 (2.5) BW: 1131 (284)	*S*. *boulardii* vs. no probiotic	Once daily from the time of initiation of enteral feeds, until discharge	NEC ≥ Stage 2	Severe 12/135	Severe 14/136	20/135	21/136
Dilli 2015^47^	GA <32 wk and BW <1500 g	n = 100 GA: 28.8 (1.9) BW: 1236 (212)	n = 100 GA: 28.2 (2.2) BW: 1147 (271)	*B*. *lactis* vs. placebo	From d 8 of life, once daily until discharge or a maximum of 8 wks	NEC ≥ Stage 2	Severe 0/100	Severe 3/100	8/100	13/100
Jacobs 2013^48^	GA <32 wk and BW <1500 g	n = 548 GA: 27.9 (2.0) BW: 1063 (259)	n = 551 GA: 27.8 (2.0) BW: 1048 (260)	*B. infantis* + *S. thermophilus* + *B. lactis* vs. placebo	From enteral feed ≥6 mL/day until discharge or term corrected age.	LOS	Severe 28/548	severe 30/551	72/548	89/551
Manzoni 2006^49^	BW <1500 g	n = 39 GA: 29.6 (5.0) BW: 1212 (290)	n = 41 GA: 29.3 (4.0) BW: 1174 (340)	*L*. *rhamnosus* GG vs. no probiotic	From d 3 of life, for 6 wks or until discharge	Enteric fungal colonization	Any 16/39	Any 18/41	19/39	22/41
Manzoni 2009^53^	BW <1500 g	n = 151 GA: 29.8 (2.8) BW: 1138 (253)	n = 168 GA: 29.5 (3.2) BW: 1109 (269)	*L*. *rhamnosus* GG + lactoferrin vs. placebo	From d 3 of life, for 6 wks or until discharge	LOS	Severe 13/151	Severe 19/168	7/151	29/168
Roy 2014^50^	GA <37 wk and BW <2500 g	n = 56 GA: 32.0 (2.0) BW: 1192 (341)	n = 56 GA: 32.2 (2.0) BW: 1069 (365)	*L*. *acidophilus* + *B*. *longum* + *B*. *bifidum* + *B*. *lactis* vs. placebo	Commenced within 72 h of birth for 6 wks or until discharge	Enteric fungal colonization	Any 14/56	Any 10/56	31/56	42/56
Sari 2012^51^	GA <33 or BW <1500 g	n = 86 GA: 29.7 (2.5) BW: 1241 (264)	n = 88 GA: 29.8 (2.3) BW: 1278 (273)	*L. sporogenes*, vs. no probiotic	From first enteral feeds until discharge	Growth and neurodevelopment at 18–22 months	Severe 5/86	Severe 4/88	24/86	19/88
Totsu 2014^52^	BW <1500 g	n = 153 GA: 28.6 (2.9) BW: 1016 (289)	n = 130 GA: 28.5 (3.3) BW: 998 (281)	*B. bifidum* vs. placebo	Commenced within 48 h of birth and continued until discharge	Day when enteral feed exceeding 100 mL/kg/d	Severe 20/153	Severe 25/130	6/153	10/130

BW: birth weight; GA: gestational age; Pr: probiotics Co: control; ROP: retinopathy of prematurity; LOS: late onset sepsis. Data of GA (weeks) and BW (grams) in the sample size column are expressed as mean (SD).

**Table 2:** Assessment of the risk of bias of the studies.

Study	Random Sequence Generation	Allocation Concealment	Blinding of Participants and Personnel	Blinding of Outcome Assessment	Incomplete Outcome Data	Selective Reporting	Other Bias
Al Hosni 2011	UR	UR	LR	LR	LR	LR	LR
Chou 2008	LR	LR	LR	LR	LR	LR	LR
Costaloe 2016	LR	LR	LR	LR	LR	LR	LR
Demirel 2013	LR	LR	LR	LR	LR	LR	LR
Dilli 2015	LR	LR	LR	LR	LR	LR	LR
Fujii 2006	UR	UR	HR	UR	LR	UR	UR
Jacobs 2013	LR	LR	LR	LR	LR	LR	LR
Manzoni 2006	LR	UR	LR	LR	LR	LR	LR
Manzoni 2009	LR	UR	LR	LR	LR	LR	LR
Roy 2014	LR	LR	LR	LR	LR	LR	LR
Saengtawesin 2014	UR	UR	HR	HR	LR	LR	UR
Sari 2012	LR	LR	LR	LR	LR	LR	LR
Totsu 2014	LR	UR	LR	LR	LR	LR	LR

LR: low risk; UR: unclear risk; HR: high risk. The studies of Fujii *et al*. and Saengtsawesin *et al*. were excluded from the meta-analysis due to the presence of unclear or high risk of bias in more than three domains.

**Figure 2:**
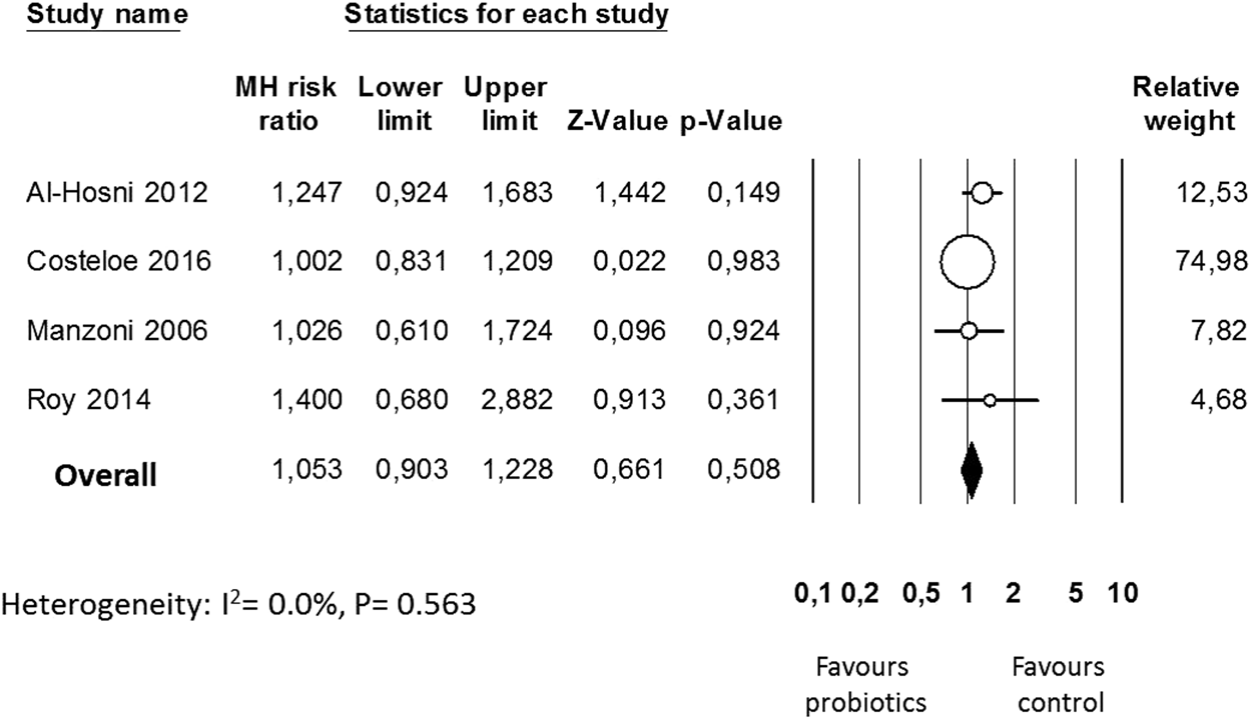
Forest plot: Probiotic supplementation and risk of any stage retinopathy of prematurity. Fixed effects model.

Although not clearly specified in the studies, it was assumed that data on ROP referred to the eye with the higher disease severity. Four studies^43,45,49,50^ reported data on any stage ROP and neither the individual studies nor the meta-analysis could detect a significant effect of probiotic supplementation (RR 1.053, 95% CI 0.903 to 1.228, P = 0.508, Fig. 2). The use of a random effects model instead of a fixed effect model did not significantly affect the results of the meta-analysis (RR 1.053, 95% CI 0.903 to 1.228, P = 0.508). In sensitivity analyses, excluding one study at a time, the summary RR ranged from 1.025 (95% CI 0.864–1.217, P = 0.774), when the study of Al Hosni *et al*.^43^ was excluded, to 1.206 (95% CI 0.932–1.562, P = 0.155), when the study of Costeloe *et al*.^45^ was excluded. The study of Roy *et al*.^50^ included larger infants than the other 3 studies (Table 1). However, when this study was excluded overall results were not substantially affected (RR 1.036, 95% CI 0.886–1.212, P = 0.658). Publication bias for the outcome of any stage ROP was not assessed due to the low number of studies.

**Figure 3:**
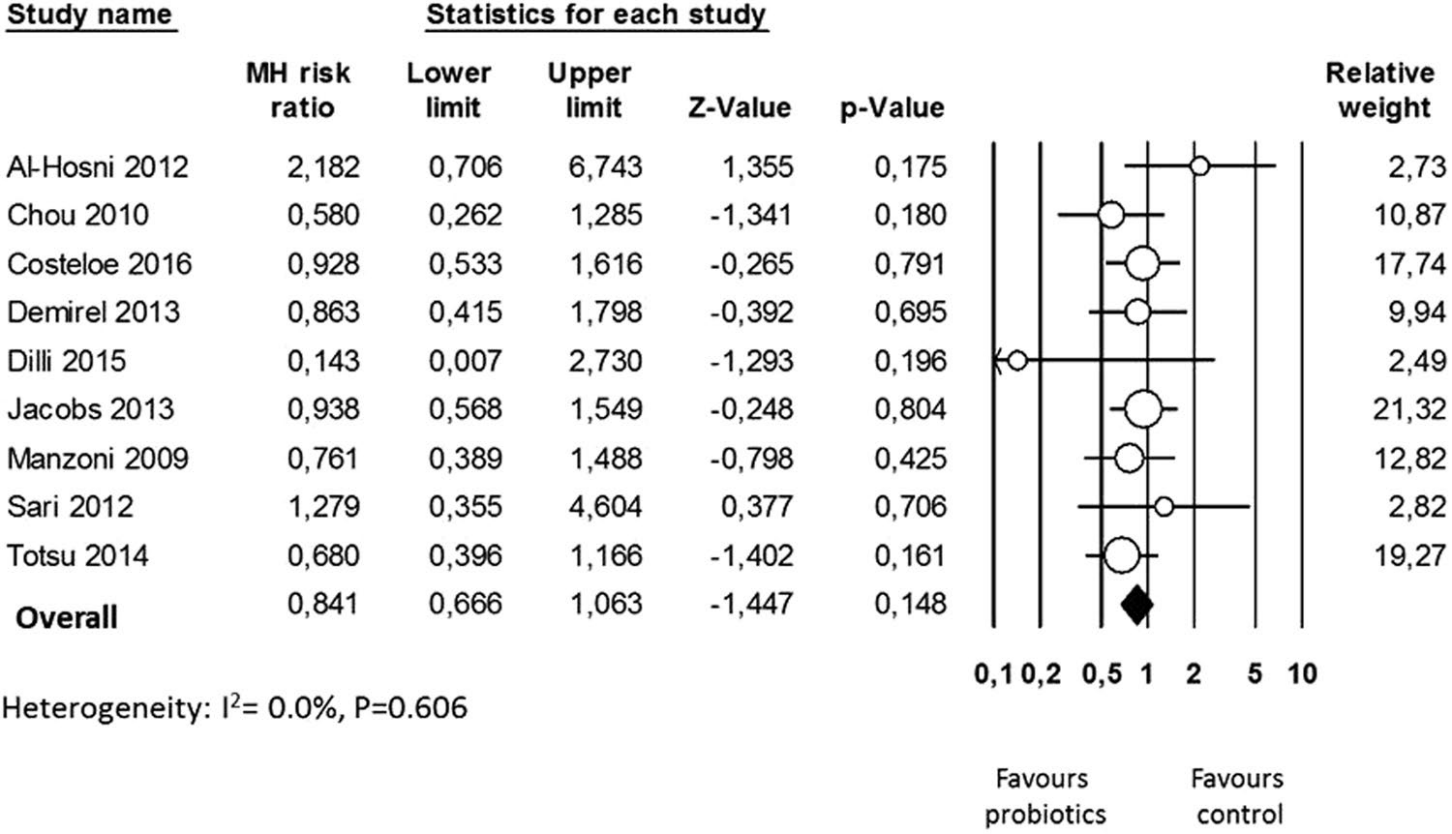
Forest plot: Probiotic supplementation and risk of severe retinopathy of prematurity. Fixed effects model.

**Figure 4:**
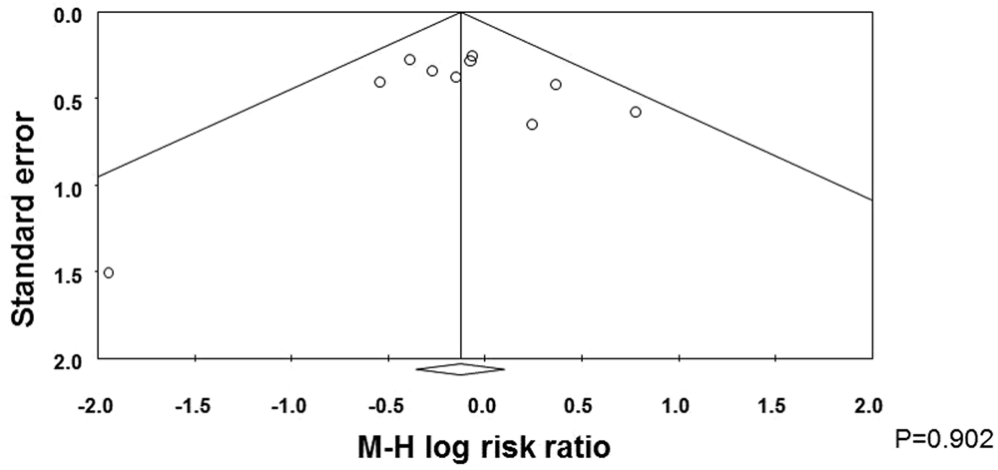
Funnel plot assessing publication bias for severe ROP.

Nine studies^43–48,51–53^ reported data on severe ROP and neither the individual studies nor the meta-analysis could detect a significant effect of probiotic supplementation (RR 0.841, 95% CI 0.666 to 1.063, P = 0.148, Fig. 3). The use of a random effects model instead of a fixed effect model did not significantly affect the results of the meta-analysis (RR 0.844, 95% CI 0.666 to 1.070, P = 0.160). Neither inspection of the funnel plot nor formal assessment using Egger’s test showed any evidence of publication bias in this analysis (Fig. 4). In sensitivity analyses, excluding one study at a time, the summary RR ranged from 0.808 (95% CI 0.634–1.029, P = 0.084), when the study of Al Hosni *et al*.^43^ was excluded, to 0.889 (95% CI 0.683–1.157, P = 0.380), when the study of Totsu *et al*.^52^ was excluded. In addition, exclusion of the analysis of the 3 studies^43,52,53^ with an unclear risk of allocation concealment bias did not substantially affect the results of the meta-analysis (RR 0.849, 95% CI 0.633–1.138, P = 0.273). Finally, exclusion of the study of Dilli *et al*.^47^, which reported a lower rate of ROP than the other studies, did not significantly affect the effect size (RR 0.849, 95% CI 0.633–1.138, P = 0.273).

**Figure 5:**
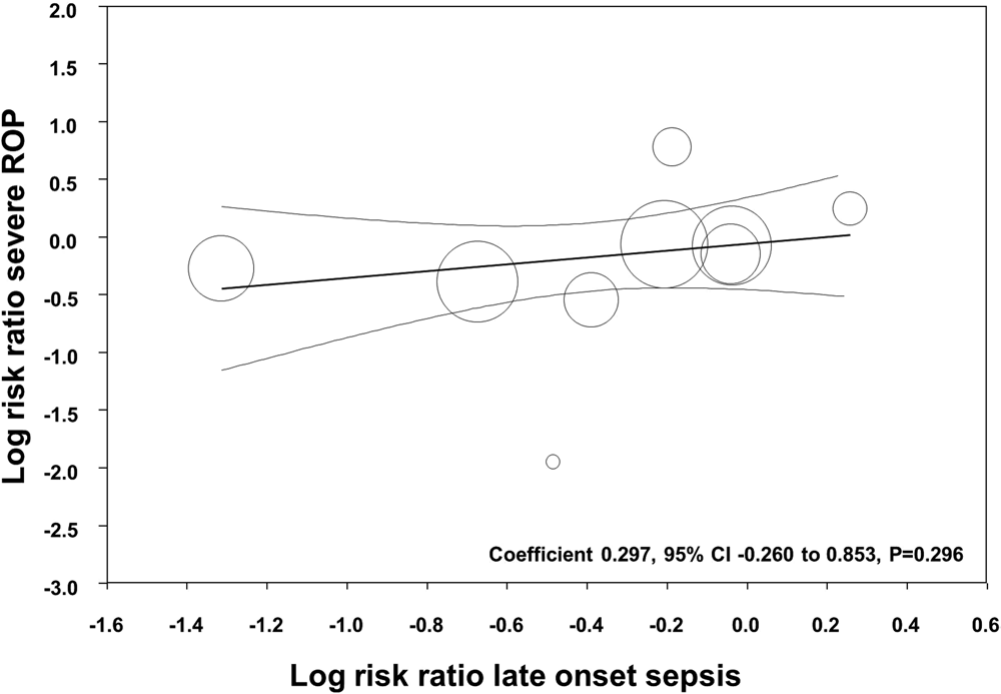
Meta-regression of the relationship between the effects of probiotics on late onset sepsis and severe ROP.

All the included studies reported data on LOS and, when pooled, we observed a significant reduction of this outcome in the probiotics group (RR 0.807, 95% CI 0.705 to 0.924, P = 0.010). This significant reduction of LOS with probiotics was also observed when the 9 studies^43–48,51–53^ reporting on severe ROP were pooled (RR 0.809, 95% CI 0.692 to 0.946, P = 0.008). We performed meta-regression analyses (methods of moments) in order to investigate the possible correlation between the effect size for LOS and the effects size for severe ROP. As shown in Fig. 5, meta-regression could not detect a statistically significant correlation between the reduction in LOS produced by the probiotics and the effect size for severe ROP. In addition, meta-regression could not detect any significant effect of the type of probiotic on the effect size for severe ROP (*Lactobacillus* yes/no: coefficient −0.048, 95% CI −0.587 to 0.473, P = 0.856; *Bifidobacterium* yes/no: coefficient 0.019, 95% CI −0.519 to 0.557, P = 0.945; multi-strain products yes/no: coefficient 0.141, 95% CI −0.635 to 0.354, P = 0.577).

Other potential moderator variables tested by meta-regression were duration (in weeks) of the supplementation with probiotics (coefficient 0.040, 95% CI −0.079 to 0.158, P = 0.515), supplementation with probiotics ≤7 weeks (yes/no; coefficient −0.315, 95% CI −0.832 to 0.203, P = 0.233), mean GA of the studied population (coefficient per week: −0.181, 95% CI −0.447 to 0.087, P = 0.185), mean BW of the studied population (coefficient per 100 g: −0.154, 95% CI −0.478 to 0.170, P = 0.351), total number of included infants (coefficient per 100 infants 0.016, 95% CI −0.040 to 0.071, P = 0.583), publication year (coefficient 0.017, 95% CI −0.092 to 0.126, P = 0.754), percentage of cesarean section (coefficient −0.028, 95% CI −0.027 to 0.021, P = 0.816), percentage of PROM (coefficient −0.004, 95% CI −0.048 to 0.040, P = 0.855), percentage of use of antenatal corticosteroids (coefficient 0.006, 95% CI −0.008 to 0.020, P = 0.390), and percentage of use of antenatal antibiotics (coefficient 0.001, 95% CI −0.021 to 0.019, P = 0.929). Due to the low number of studies reporting on maternal infection and percentage of use of exclusive maternal milk (Supplementary Table 1), meta-regression for these two moderators was not performed.

## Discussion

Probiotic supplementation in preterm infants is one of the most studied interventions in neonatal medicine^16,19,26,29–34,56^. However, to the best of our knowledge, this is the first meta-analysis assessing the effect of probiotics on the development of ROP. Despite a reduced risk of LOS, our study could not demonstrate any significant effect of probiotic supplementation on the risk of developing ROP. The validity of our meta-analysis is potentially compromised as the included trials were highly variable with regard to enrolment criteria (i.e., BW and GA), timing, dose, and formulation of probiotic used. Moreover, ROP was not the primary outcome in any of the trials and the definition of severe ROP varied among the studies. In addition, separate data on the population with the highest risk of ROP (i.e., extremely preterm or extremely low BW infants) could not be retrieved.

Increased susceptibility to infections in the preterm infant is due to functional defects of both innate and adoptive immunity combined with prolonged hospitalization, and frequent need for invasive procedures^57,58^. As reviewed by Lee & Dammann^3^, the effects of infection and inflammation in the pathogenesis of ROP may be direct, indirect, or both. Proinflammatory cytokines may exert a direct effect on retinal angiogenesis or sensitize the developing retina to the effects of oxygen, or other stressors. On the other hand, the circulatory instability and fluctuation of oxygen saturation following sepsis may affect the retinal perfusion and lead to increased retinal injury, particularly during the second phase of pathological vessel growth.

Despite the pathogenic role of sepsis in the development of ROP, there is very little literature published on reducing the risk of infection in preterm neonates and its effect on ROP incidence^1^. As mentioned in the introduction, only the effect of fungal infection prophylaxis with fluconazole has been systematically analyzed^1^. Our study is the largest meta-analysis to date investigating the effects of a strategy of infection reduction on the development of ROP. We expected that an intervention, such as probiotic supplementation, with proven efficacy in reducing LOS, would also affect the rate of ROP. In fact, when the 11 studies included in our meta-analysis were pooled, it was observed a reduction of the risk of LOS among the infants receiving probiotics (RR 0.81, 95% CI 0.71 to 0.92). Interestingly, this RR was very similar to the one reported by Rao *et al*. in their meta-analysis on probiotics and LOS (RR 0.86, 95% CI 0.78 to 0.94)^26^. It should be noted that the meta-analysis of Rao *et al*. included 37 RCTs (i.e., the 11 studies of the present meta-analysis plus 26 studies that did not report on ROP). Therefore, our sample of 11 studies appears to be representative of the effects of probiotics on LOS reduction in preterm infants.

We performed a meta-regression analysis in order to investigate whether the studies achieving a higher rate of protection against LOS also achieved a higher rate of protection against severe ROP. Meta-regression is a statistical technique which examines the relationship between continuous or categorical moderators and the size of effects observed in the studies^41,42^. Thus, meta-regression allows for the exploration of more complex questions than does traditional meta-analysis. In our study, meta-regression could not show any significant correlation between the RR for LOS and the RR for severe ROP. This suggests that the reduction in sepsis rate did not translate into a reduction of ROP. Nevertheless, it should be taken into account that a robust conclusion from meta-regression would require a larger number of included studies^41,42^.

Since probiotic supplementation in RCTs is not a homogeneous intervention, the choice of probiotic strain(s) is crucial and meta-analyses may be misleading with the risk that generalized conclusions are erroneously extrapolated to other probiotics^16,59^. Furthermore it is unclear whether multi-strain products are more effective than single strain products^16^. Separate meta-analyses of the effects of well-defined individual, single strain or multiple-strain probiotic preparations appears to be more appropriate but the important heterogeneity of the RCTs makes this approach unfeasible^16^. In the present study, meta-regression could not detect any significant effect of the type of probiotic (*Lactobacillus, Bifidobacterium* or multi-strain products) on the effect size for severe ROP. Of note is that the largest trial published so far^45^ was negative for all clinical outcomes, including ROP, but the study product (*B breve* BBG-001) had never been reported to have any clinical effect in neonates^59^. Therefore, more studies to address the optimal probiotic preparation, dosing, and duration of therapy are still needed in head to head comparative studies rather than placebo controlled trials^29^. These studies should compare strains that have been reported to be safe and effective in previous trials^59^.

It has been suggested that the type of milk (human or formula) that infants receive modifies the effect of probiotics^60,61^. Interestingly, in a recent meta-regression analysis, Thomas *et al*. showed that the effectiveness of probiotics in reducing NEC was higher in cohorts with increased rates of exclusive human milk^61^. Unfortunately, most studies included in the present analysis lacked information on type of feeding and, therefore, we were not able to stratify by type of milk feeding. Besides the use of human milk, other prenatal and postnatal factors such as maternal infections, delivery mode (vaginal delivery vs. cesarean section), or antibiotic use may shape the initial bacterial inoculum of the newborn and further configure the microbiome during early life^62,63^. This may interfere with the effects of probiotics. Thomas *et al*. showed, through meta-regression, that probiotics were more effective in preventing NEC in cohorts where lower proportions of mothers received antenatal corticosteroids^61^. In contrast, the rate of cesarean section did not significantly correlate with the effect of probiotics on NEC^61^. The present meta-regression analysis could not demonstrate a significant correlation between the rate of antenatal corticosteroids, antenatal antibiotics, PROM, or cesarean section and the effect of probiotics on ROP. However, as mentioned above, our meta-regression is limited by the low number of studies.

The issue of whether it is time to change practice and adopt the use of probiotics as a standard of care in preterm infants remains a hot topic for neonatologists. While some advocate a change in practice based on significant reduction in time to achieve full enteral feeding, severe NEC, LOS, and all-cause mortality^17,19,26,29,38,61^, others have raised concerns about the methodology of many of the published and advocate for waiting until further data on efficacy and safety in extremely preterm infants are available^16,62–65^. The evidence summarized in this meta-analysis suggests that, despite being effective in reducing LOS, probiotic supplementation does not affect the incidence of ROP in preterm infants. Nevertheless, further studies addressing this issue are needed to confirm our findings^66–68^.

## Electronic supplementary material

Supplementary Table 1
