# Supplementary material for: Probiotic supplementation in preterm infants does not affect the risk of retinopathy of prematurity: a meta-analysis of randomized controlled trials
Source: Sci Rep. 2017 Oct 12;7:13014. doi: 10.1038/s41598-017-13465-2 (PMC5638943; doi:10.1038/s41598-017-13465-2)
Supplement: Supplementary file 1 — Supplementary Table 1 [file 41598_2017_13465_MOESM1_ESM.pdf]

Probiotic supplementation in preterm infants does not affect the risk of retinopathy of prematurity: a meta-analysis of randomized controlled trials

Giacomo Cavallaro , Eduardo Villamor-Martínez, Luca Filippi, Fabio Mosca, and Eduardo Villamor.

Supplementary Table 1. Additional maternal and neonatal characteristics of the studies.

**Study**	**Antenatal antibiotics**		**Antenatal steroids**		**PROM**		**Maternal infection**		**Cesarean section**		**Exclusive maternal milk**	
	RR (95% CI)	% total	RR (95% CI)	% in study	RR (95% CI)	% in study	RR (95% CI)	% in study	RR (95% CI)	% in study	RR (95% CI)	% in study
**Al Hosni 2011**	NA	NA	1.00 (0.84 - 1.18)	85/101 (84%)	NA	NA	NA	NA	0.75 (0.51 - 1.10)	52/101 (51%)	NA	NA
**Chou 2008**	NA	NA	1.1 (0.92 - 1.31)	190/301 (63%)	1.19 (0.81 - 1.77)	76/301 (25%)	NA	NA	1.08 (0.88 - 1.32)	167/301 (55%)	NA	NA
**Costeloe 2016**	0.99 (0.85 - 1.15)	446/1310 (34%)	0.97 (0.94 - 1)	1187/1310 (91%)	0.93 (0.78 - 1.11)	358/1310 (27%)	1.11 (0.84 - 1.47)	168/1228 (14%)	0.99 (0.89 - 1.10)	690/1309 (53%)	1.00 (0.89 - 1.12)	606/1310 (46%)
**Demirel 2013**	NA	NA	1.04 (0.83 - 1.29)	146/271 (54%)	1.10 (0.50 - 2.4)	23/271 (8%)	NA	NA	0.94 (0.83 - 1.05)	218/271 (80%)	NA	NA
**Dilli 2015**	0.43 (0.11 - 1.61)	10/200 (5%)	1.08 (0.84 - 1.38)	110/200 (55%)	1.00 (0.49 - 2.05)	26/200 (13%)	NA	NA	0.95 (0.65 - 1.37)	72/200 (36%)	1.10 (0.84 - 1.45)	101/200 (51%)
**Jacobs 2013**	0.98 (0.86 - 1.1)	532/1099 (48%)	1.01 (0.97 - 1.05)	1002/1099 (91%)	NA	NA	0.98 (0.67 - 1.45)	95/1099 (9%)	0.96 (0.88 - 1.04)	736/1099 (67%)	NA	NA
**Manzoni 2006**	0.88 (0.69 - 1.12)	62/80 (78%)	0.97 (0.73 - 1.28)	57.025/80 (71%)	NA	NA	NA	NA	1.08 (0.79 - 1.46)	53.95/80 (67%)	NA	NA
**Manzoni 2009**	0.97 (0.86 - 1.1)	242/319 (76%)	0.94 (0.82 - 1.08)	227/319 (71%)	NA	NA	NA	NA	1.02 (0.91 - 1.14)	249/319 (78%)	0.96 (0.63 - 1.46)	69/319 (22%)
**Roy 2014**	0.77 (0.53 - 1.14)	55/112 (49%)	1.04 (0.88 - 1.24)	92/112 (82%)	0.63 (0.34 - 1.17)	31/112 (28%)	2.50 (0.51 - 12.35)	7/112 (6%)	1.09 (0.91 - 1.31)	90/112 (80%)	NA	NA
**Sari 2012**	NA	NA	0.64 (0.41 - 1)	57/174 (33%)	1.02 (0.45 - 2.33)	20/174 (11%)	NA	NA	0.91 (0.76 - 1.1)	125/174 (72%)	NA	NA
**Totsu 2014**	NA	NA	1.30 (1.06 - 1.59)	167/283 (59%)	NA	NA	NA	NA	0.75 (0.64 - 0.88)	194/283 (69%)	NA	NA

CI: confidence interval; NA: information not available; RR: risk ratio. RR>1 indicates higher risk of the characteristic in the probiotic group.
